# Difference in the Catalytic Activity of Atoms in the Corners and at the Edges of Gold Nanoparticles: Hydrogen Isotope Exchange Reaction

**DOI:** 10.3390/ijms252212022

**Published:** 2024-11-08

**Authors:** Evgeny V. Abkhalimov, Boris G. Ershov

**Affiliations:** A.N. Frumkin Institute of Physical Chemistry and Electrochemistry, Russian Academy of Science, Leninsky pr. 31-4, 119071 Moscow, Russia; ershov@ipc.rssi.ru

**Keywords:** active site, turnover frequency, low-coordination atoms, coordination number

## Abstract

The goal of this work is to investigate the catalytic activities of low-coordination atoms located in gold nanoparticles. Gold nanoparticles with sizes from 0.7 to 40 nm deposited on γ-Al_2_O_3_ were used as a catalyst. Synthesized gold nanoparticles and prepared catalysts were characterized by HRTEM, SEM, XRD, DLS, and UV-Vis spectroscopy. The specific activity of gold nanoparticles towards the isotope exchange reaction at 77 K was studied as a function of nanoparticle size. The catalytic activity increases significantly when the particle size is less than 3 nm. The activities of low-coordination gold atoms located at the edges and in the corners are markedly different. Corner atoms (CN = 6) are more than 40 times more active in the reaction of hydrogen isotope exchange than edge atoms (CN = 7). TOF for atoms with coordination numbers CN = 6 and CN = 7 are 0.258 ± 0.020 and 0.006 ± 0.001 molecules site^−1^ s^−1^, respectively. An equation was proposed for the dependence of the catalytic activity of the reaction on the particle size, the number of atoms on the surface, and their activity.

## 1. Introduction

Nanoscale gold exhibits unique properties that are not present in bulk metal. Specifically, it has the ability to catalyze numerous reactions [1,2,3,4,5]. In most cases, catalytic activity increases as particle size decreases, with the effect being particularly evident in particles ≤ 5 nm [6,7,8,9,10]. This can be attributed to the metal-support interfaces playing a specific role [9,11,12], as well as the quantum-size effect [13,14,15], surface defects [16,17], and the presence of low-coordination corner and edge atoms in gold nanoparticles. The number of these atoms increases significantly, as particle size decreases [18,19,20,21,22,23,24,25].

In hydrogenation reactions of organic compounds, the dissociation of the hydrogen molecule into atoms is the rate-limiting step [26]. The efficiency of dissociative adsorption of hydrogen on gold nanoparticles determines the rate of catalytic reactions involving hydrogen. Homomolecular hydrogen isotope exchange through the reaction H_2_ + D_2_ ↔ 2HD is an ideal model for studying the mechanism and kinetics of dissociative hydrogen adsorption. This exchange only occurs through the splitting of the hydrogen molecule into atoms and is not complicated by any other reactions. Previously, it was found that homolytic hydrogen dissociation proceeds on the surface of gold nanoparticles (Au-Al_2_O_3_) at the corner and edge atoms [18]. Recently, it was found that the H only binds to the edge and corner sites of the AuNPs [27]. It has been shown by the XAS method that hydrogen dissociation proceeds on both substrate-bound and substrate-unbound NPs [28]. During hydrogen chemisorption on small gold clusters (1–2 nm) in solution, the appearance of a plasmonic absorption peak is observed in the optical spectrum [28], and for larger NPs there is a shift in the position of the maximum of the SPR absorption band [25,29]. These effects are due to the formation of anti-bonding 5d Au-H 1s and doping of the gold sp-band by electrons of absorbed hydrogen atoms [28]. Studies have shown that gold nanoparticles supported on γ-Al_2_O_3_ catalyze homomolecular hydrogen isotope exchange at low temperatures down to 77 K [18,19,20,21]. The specific catalytic activity increases approximately three orders of magnitude as particle size decreases from 40 nm to 0.7 nm. Empirical equations that relate the catalytic activity of particles to particle size and the concentration of low-coordination gold atoms have been derived [19]. It is possible that single catalytic sites (gold atoms at the edges and corners) responsible for dissociative hydrogen adsorption have varying catalytic activities. In other words, they differ significantly in their ability to activate the splitting of hydrogen molecules into atoms. Given the importance of studying the mechanism and kinetics of dissociative hydrogen adsorption and the nature of catalytic sites for hydrogenation, it is crucial to verify this assumption and the possibility of controlling the rate of catalytic synthesis of organic compounds involving hydrogen.

The present study aims to investigate the difference between the catalytic activities of low-coordination edge and corner atoms of gold nanoparticles. We measured the specific activity of gold nanoparticles supported on γ-Al_2_O_3_ with respect to the isotope exchange reaction, depending on particle size, and compared the obtained activity with the number of surface low-coordination atoms in cuboctahedral gold nanoparticles with coordination numbers (CNs) ≤ 6 (corners) and CN = 7 (edges). The reaction was conducted at 77 K to suppress any other possible thermally activated reactions. Our results confirm the assumption that corner and edge atoms of nanoparticles significantly differ in their catalytic activity towards the isotope exchange of hydrogen.

## 2. Results and Discussion

**Figure 1:**
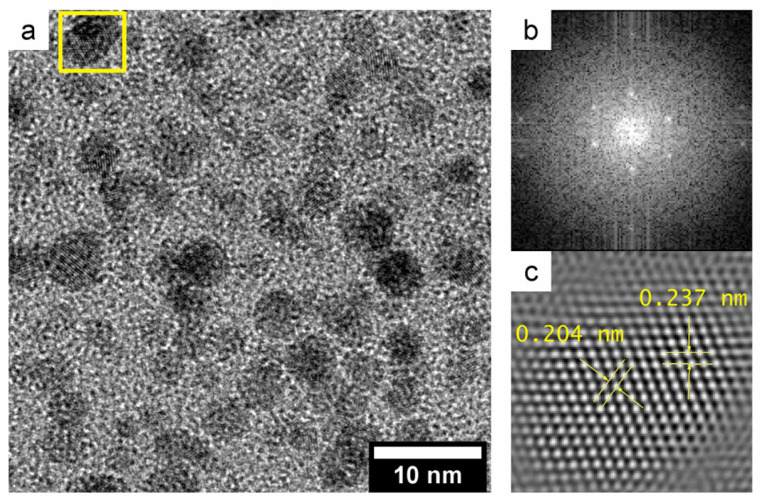
TEM (**a**), FFT of yellow rectangle in image (**a**). (**b**) and inverse FFT of light spot in image (**b**). (**c**) images of 4.6 nm Au NPs.

A typical TEM image of synthesized gold nanoparticles is shown in Figure 1a. As can be seen, the particles are spherical. NPs are crystalline and have lattice spacing of 2.37 Å and 2.04 Å of 111 and 200 lattice, respectively. The average size of the NPs is 4.6 ± 0.8 nm. Other TEM images of gold nanoparticles are exhibited in Figure S1. The average sizes are from 0.7 to 40.1 nm. During the preparation of catalysts, gold nanoparticles were heated. This could lead to a change in their sizes. However, we have previously shown that no significant changes in the nanoparticle sizes were observed at temperatures below 573 K [19]. Also, molecular dynamics calculations have shown that coagulation does not occur [30] and the particles remain in their initial shapes in the low-temperature region [31].

The crystal structure of the gold nanoparticles was determined with SAED and XRD analysis. Figure S2 shows the electron diffraction pattern. The rings of 111, 200, 220, and 311 planes are clearly seen in the pattern. These rings correspond to the formation of metallic gold NPs with a face-centered cubic lattice. The calculated values of the lattice parameter and interplanar spacing confirm the formation of gold NPs (See Table S1).

**Figure 2:**
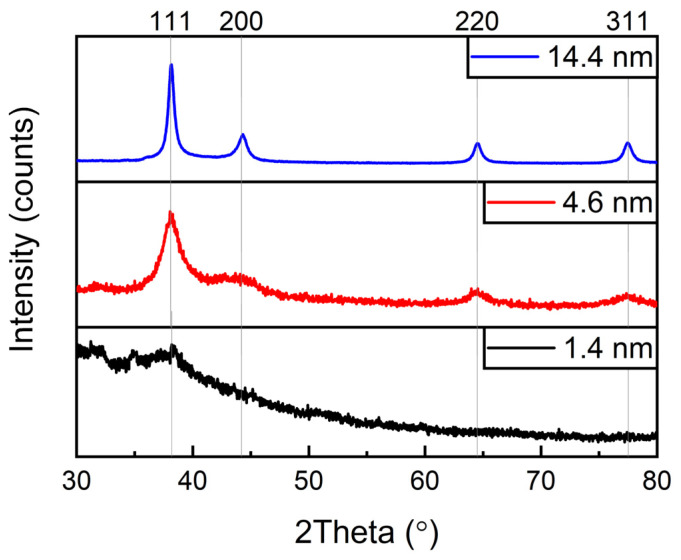
XRD spectra of Au NPs of three different sizes.

The crystal structure of NPs was also examined by powder XRD, as shown in Figure 2. It can be seen that the XRD spectra exhibit evident diffraction peaks at ca. 38.2°, 44.2°, 64.5°, and 77.5°, which could be indexed well to the 111, 200, 220, and 311 facets of the typical fcc phase of gold (JCPDS no. 04-0784), respectively [32,33]. It should be noted that particles smaller than 2 nm are amorphous, and the spectra show only one broad reflection at 38.2° [34]. The electron diffraction and XRD data are in good agreement.

The isotope exchange reaction does not proceed on the γ-Al_2_O_3_ support or on the bulk gold under ambient conditions. Meanwhile, supported nanosized gold particles of any size from 0.7 nm to 40.1 nm catalyze the exchange reaction. In other words, the nanosized state of the metal accounts for the ability to initiate the dissociative adsorption of hydrogen, followed by the formation of HD molecules via recombination of H and D atoms [18,19,35]. The isotope exchange follows first-order kinetics. The specific catalytic activity (*K_sp_*) was expressed by Equation (1) as follows:

(1) $$K_{sp}=\frac{K_{0}N_{T}}{S_{H}},$$

where *K*_0_—the first-order reaction rate constant, s^−1^;

*N_T_*—the number of hydrogen atoms in the reaction volume at a measured temperature, molecules;

*S_H_*—the active surface area, cm^−2^.

The rate constants, active surface, and activation energy of the Au@Al_2_O_3_ catalysts for particles of different sizes are presented in Table 1.

**Figure 3:**
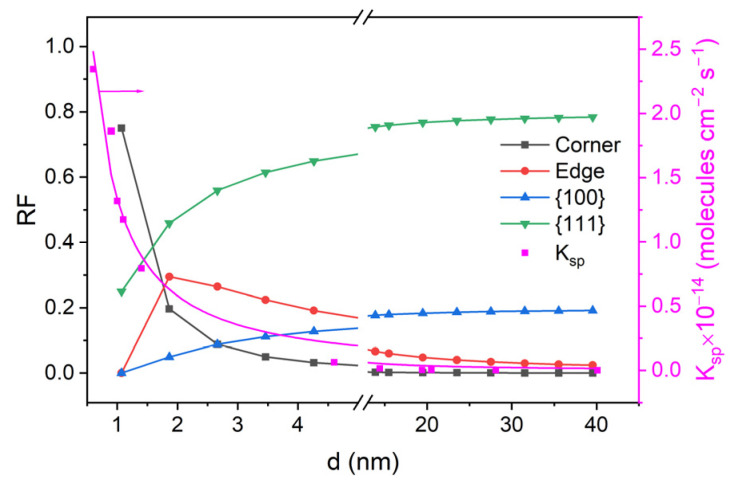
Size dependences on the specific catalytic activity and the relative frequencies of the edge and corner gold atoms.

Figure 3 shows the variation in *K_sp_* as a function of particle size, illustrating the increase in the specific activity with decreasing size. The plot consists of two sections: smooth growth of the catalytic activity as the particle size decreases from 40 nm to approximately 5 nm (Figure 3). A similar dependence was previously observed [12,36,37,38]. The slow growth section is replaced by a sharp increase in the activity for particles less than 4 nm (Figure 3, magenta curve). Hence, the size of gold particles proves to be the factor responsible for the specific catalytic activity. It follows from Figure 3 that the specific catalytic activity *K_sp_* at 77 K drastically and exponentially decreases by a factor of approximately 2500 as the particle size increases from 0.7 nm to 40 nm. This is caused by a sharp increase in the concentration of low-coordination gold atoms on the surface of gold nanoparticles. It should be noted that, when the particle size is less than 1.1 nm, all surface atoms are low coordinated (CN ≤ 6).

In this case, the number of atoms per nanoparticle begins to play a significant (decisive) role. This is explained by the fact that the number of surface atoms becomes less than their content in the smallest cuboctahedron (d ≈ 1.07 nm). In contrast to large particles, for which the number of corner atoms remains constant at sizes larger than 1.1 nm, for small clusters at sizes smaller than 1.1 nm the number of surface and thus low-coordinated atoms decreases. Also, for such particles, interface atoms (perimeter atoms) between NPs and the support may begin to play a significant role, which can also exhibit high catalytic activity [12]. However, we have not been able to determine the effect of such atoms on the rate constant. Previously, it was found that the binding of the oxygen atom on the TiO_2_ substrate (rutile) played an essential role and promoted the dissociation of molecular hydrogen at the nearest corner and edge atoms on the surface of small gold clusters [12,39]. However, there are no unambiguous data on the influence of such atoms in the case of gold nanoparticles deposited on γ-Al_2_O_3_ substrate. It is worth noting that a significant influence of perimeter atoms was observed for the process in the temperature region of 350–450 K [12]. The values of activation energies of the process were close to those obtained by us for the high-temperature region (See Table 1). The temperature dependences exhibit characteristic inflections that partition the curves into low-temperature (LT) and high-temperature (HT) regions with disparate activation energies. This can be attributed to the differing reaction mechanisms that are operative in these temperature regions. In the temperature range of 77 to 300 K, the hydrogen atom exchange is observed to be almost independent of temperature (*E_a_*~0–2 kJ/mol), with the reaction rate apparently limited by the formation of H atoms upon dissociative adsorption of a H_2_ on gold NPs. The rate of this process is found to depend substantially on the particle size. The most notable distinction in the catalytic properties is evident at 77 K. At this temperature, the specific catalytic activity (*K_sp_*) exhibits a decrease of approximately 780-fold as the particle size increases from 0.7 nm to 40.1 nm (Figure 3). Consequently, as the nanoparticle size increases, the catalytic activity of the nanoparticles towards the H_2_-D_2_ exchange markedly declines. As has been previously observed, no hydrogen adsorption or isotope exchange is detected in the bulk metal. In the high-temperature region (above 300 K), the reaction proceeds with a high activation energy (>10 kJ/mol). In this region, *K_sp_* is virtually independent of particle size due to the rate-limiting role of thermal dissociation of the hydrogen molecule in the isotope exchange process, resulting in chemisorbed hydrogen atoms on the metal surface.

Within the framework of statistical cuboctahedron model [23], the percentages of edge and corner atoms versus the total number of atoms on the nanoparticle surface (*RF*) were calculated. The choice for the calculations of the cuboctahedral shape particle was made because this shape is the most energetically stable. Also, as shown earlier, the concentration of surface low-coordination gold atoms weakly depends on temperature [40]. The dependences of the numbers of such atoms on the particle size are also shown in Figure 3. Comparison of dependences of the *RF* and *K_sp_* values on the gold nanoparticle size demonstrates the following: the section of slow increase in the constant following the decrease in the particle size from 40 nm down to approximately 3–4 nm is rather well correlated with the increase in the number of edge gold atoms, while the fast growth section for ≤ 1.5 nm particles is correlated with the increase in the number of corner atoms. Thus, the catalytic activity of gold nanoparticles shows quite a definite relationship with positions of the gold atoms on the surface, that is, at the edges and in the corners of particles. Calculations show that, for nanoparticle sizes ranging from 40 nm to about 15 nm, edge atoms predominate among the low-coordinated atoms on their surface. For these “large” particles (≥15 nm) their relative frequency is more than 90%. The percentage of corner gold atoms in this range of particle sizes is very low, so that it can be neglected. A different situation is observed for “small” particles in the 0.7–1.5 nm range. In this case, conversely, the gold atoms located in the cluster atoms with CN = 6 predominate. Therefore, the sharp increase in the catalytic activity in the 0.7–1.5 nm range of particle size should be attributed to the presence of corner and perimeter interface gold atoms. Note that the predominance of low-coordination gold atoms with CN = 6 for <1.5 nm sizes is correlated with the loss of metallic behavior and the appearance of quasi-metallic behavior of gold particles [41,42].

The presence of particles with a predominance of low-coordination corner and perimeter interface gold atoms of a particular type can be used to evaluate their catalytic activity [43]. The increased coordination number usually causes the downward shift of the d-band center position, thus regulating the catalyst-substrate/intermediate interaction and leading to high catalytic activity [28]. Previously, the investigation of Au NPs covered with chemisorbed hydrogen using XAS spectroscopy showed that the intensity of the white line is enhanced due to the formation of unoccupied Au 5d−H 1s antibonding bands. The interaction between the H 1s orbital and Au d-band generated the bonding and antibonding states below the d-band and above the Fermi level, respectively. The formation of such antibonding bands means the presence of bonding interaction between the H 1s orbital and Au 5d band [28]. No increase in the gold CN was observed during hydrogen adsorption. Apparently, a similar phenomenon takes place in the case of low-coordinated atoms of the small gold particles studied by us.

**Figure 4:**
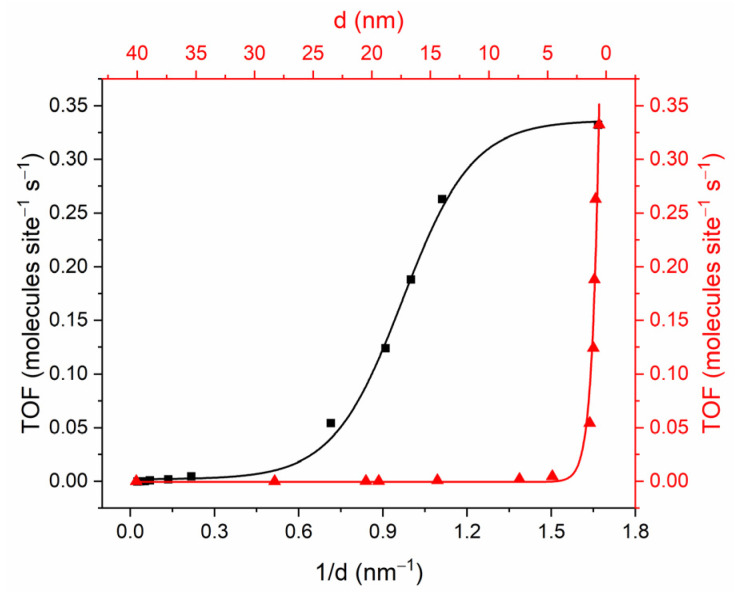
Dependence of turnover frequency based on the surface atom versus dispersion degree and the mean diameter of gold nanoparticles.

At particle sizes of 14.4 nm or greater, edge atoms represent a significant proportion of the low-coordinated gold atoms. Conversely, at particle sizes of less than 1.5 nm, corner gold atoms are the predominant sites. At these sites, gold atoms with different CNs exhibit markedly different catalytic activities with respect to hydrogen isotope exchange. Figure 4 shows the plots for the *TOFs* per surface atom versus degree of dispersion (*d*^−1^) and particle size. As can be seen, *TOF* increases exponentially with an increasing degree of dispersion. This behavior is named sympathetic structure sensitivity. Such dependence is related to the different adsorption behaviors of reactants on different crystal sites, namely on faces, corners, edges, or interfaces of the metal support on the metal NP surface, since their *RF* change significantly with particle sizes in the range of 1–10 nm, which critical important for catalysis [44,45]. It is important to note that the dependence of the catalytic activity on the Au particle size to a higher density of low-coordination atoms, the *TOF* per active atom, does not depend on the particle size. This is true for both small and large particles [37].

**Figure 5:**
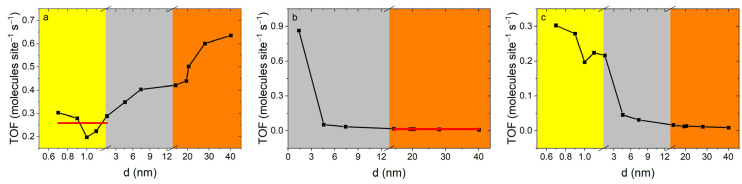
Dependence of turnover frequency vs. diameter of particles: corner atoms (**a**), edge atoms (**b**) and sum of corner and edge (**c**) atoms. Highlighted colored zones show the ratio of the number of corner and edge atoms. Yellow—*N*_c_ ≥ *N*_e_; grey—*N*_c_ ≈ *N*_e_; orange—*N*_c_ ≤ *N*_e_.

To test the hypothesis that the catalytic activity of gold nanoparticles is related to the density of active atoms located on the surface, we calculated the *TOF* dependences on the theoretical number of corner (a), edge (b), and the sum of corner and edge (c) atoms (Figure 5). If only corner atoms were active, the *TOF* calculated considering only corner atoms would be independent of size. It can be seen (Figure 5a,b red lines) that, indeed, two regions of *TOF* independence from NP size are observed. These are the particle size region of 0.7–1.4 nm, corresponding to the *TOF* with CN 6, and 14.4–40.1 nm, corresponding to the *TOF* for CN 7. If the activities of atoms with coordination numbers six and seven were the same, the TOF calculated for their sum would not depend on the particle size. However, as can be seen (Figure 5c), *TOF* is size dependent. The *TOF* for atoms with CN of six and seven are 0.258 ± 0.020 and 0.006 ± 0.001 molecules site^−1^ s^−1^, respectively. Thus, the activity of the corner atoms was found to be more than 40 times higher.

As we note before, the atoms at the nanoparticle surface can provide significantly different contributions to the catalytic activity than their fully coordinated inner atoms. The contribution of internal atoms can be neglected, since hydrogen atoms cannot diffuse into the volume of gold nanoparticles [10]. Previously obtained experimental and quantum chemical modeling data showed that for hydrogen atoms, the state above the surface of the gold cluster is energetically more favorable than the state in the bulk [46]. Also, the contribution to the properties of corner and edge atoms is very different too. In accordance with the assumption of the additive model, the dependence of the specific rate constant on the *RF* of surface atoms can be expressed as (2)

(2) $$K_{sp}=K_{e}{\times RF}_{e}+K_{c}{\times RF}_{c}+K_{100}\times {RF}_{100}{+K}_{111}{\times RF}_{111},$$

where *RF*—the proportion of the corresponding atoms in the total number of surface atoms;

*K*_*e*__,*c*,100,111_—the relative rate constant of the isotope exchange reaction for the corresponding atom.

The contribution of atoms with coordination numbers eight and nine (100 and 111 sites, respectively) to the specific rate constant is negligible. This is due to the fact that the 100 and 111 sites are not active in the hydrogen isotope exchange reaction [12]. Thus, Equation (2) can be written as Equation (3) as follows:

(3) $$K_{sp}=K_{e}{\times RF}_{e}+K_{c}{\times RF}_{c},$$

The contribution of each type of atom in due time depends on their relative fraction on the surface of the particle and depends on its size. For small sizes of gold NPs (less than 5 nm), the relative fraction (*RF*) of corner atoms (the CN ≤ 6) tends to 100% (Figure 3). In addition, the *RF* of edge atoms for NPs less than 2 nm decreases, and for clusters less than 1.1 nm (24 atoms CN = 6 and 8 atoms CN = 9), almost all surfaces consist of atoms with CN ≤ 6. In this case, the contribution of the reaction rate constant for edge atoms (*K_e_*) can be neglected, and the overall rate constant (*K_sp_*) is equal to the rate constant for corner atoms (*K_c_*) (Equation (4)).

(4) $$K_{sp}\approx K_{c}{\times RF}_{c}$$

At the same time, the rate constant can be expressed in terms of *TOF* by the following Equation (5):

(5) $$K_{sp}=TOF\times N_{a}\times N_{NP},$$

where *N_a_*—the number of low-coordinated atoms on the surface of the nanoparticle;

*N_NP_*—the number of nanoparticles in 1 cm^2^ of the catalyst.

Based on Equations (3)–(5), we obtain the following expression for the reaction rate constant, expressed in terms of the values *TOF*, *RF*, and *d* (Equation (6)):

(6) $$K_{sp}=\frac{({TOF}_{c}\times {RF}_{c}+{TOF}_{e}\times {RF}_{e})\times N_{surf}}{\pi d^{2}},$$

**Figure 6:**
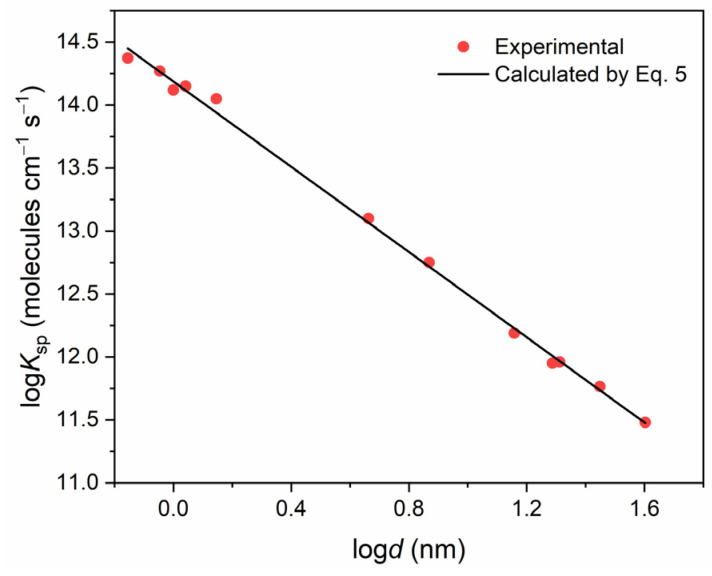
Dependence of the catalytic activity of gold nanoparticles on their size: dots—experiment and line—calculation according to Equation (5).

Using the size dependence of the relative frequencies of the edge and corner atoms (Figure 3) and the *TOF* for the edge and corner atoms, we calculated the dependence of the catalytic activity of gold nanoparticles over the range of particle sizes from 0.7 nm up to 40.1 nm, considering the contributions of the gold atoms of different types. It can be seen (Figure 6) that the resulting Equation (5) describes the experimental data well.

## 3. Materials and Methods

### 3.1. Materials

Aluminum oxide (γ-Al_2_O_3_) was obtained from by Redkinskiy Catalyst Plant. Gold(III) chloride trihydrate (AuCl_3_ 3H_2_O, ≥ 99.9%), sodium borohydride (NaBH_4_, 99%), tannic acid (C_76_H_52_O_46_, ACS Grade), tri-sodium citrate dehydrate (C_6_H_5_Na_3_O_7_ 2H_2_O, ≥99%), quercetin (C_15_H_10_O_7_ 2H_2_O, ≥95%), dioctyl sulfosuccinate sodium salt (AOT, ≥97%) all being ACS Reagent Grade chemicals purchased from Sigma Aldrich. Twice distilled water was additionally deionized with an Arium 611 setup (Sartorius AG, Göttingen, Germany) before being used for solution preparation had a specific electrical conductivity of not more than 0.056 mS cm^−1^.

### 3.2. Synthesis of Nanoparticles

Gold hydrosols containing nanoparticles of different sizes were prepared by reduction of Au(III) ions in water by various methods and in the presence of various stabilizers. Particles of 0.7 ± 0.2, 1.4 ± 0.3 nm size were prepared by the radiation chemical reduction of Au(III) ions in reverse micelles [47], while 0.9 ± 0.3, 1.0 ± 0.3, and 1.1 ± 0.3 nm particles were also obtained in reverse micelles in the presence of quercetin [48], and 4.6 ± 0.8 nm particles were prepared by photochemical reduction [49]. The particles of 7.4 ± 1.1 and 14.4 ± 2.2 nm size were synthesized using citrate in the presence of hydrogen [29], while the 19.4 ± 4.2, 28.3 ± 3.1, and 40.1 ± 5.4 nm particles were obtained by the known Turkevich method [50]. The 20.5 ± 4.5 nm particles were synthesized by reduction of Au(III) ions with tannic acid [51].

To remove the synthesis products and unbound stabilizer, the NPs were further purified. Obtained solutions were deionized three times on a 5324 centrifuge (Eppendorf, Germany) by using a regenerated cellulose centrifugal filter Amicon Ultra-0.5 (Millipore GmbH, Temecula, CA, USA) having a membrane filter with a cutoff molecular weight of 10 kDa for particles smaller than 10 nm or by centrifugation (21,000× *g*, 15 min) for particles bigger than 10 nm.

### 3.3. Preparation of Catalyst

The catalysts were manufactured by the impregnation method as follows. At first, 1 g of support was mixed in a 10 mL flask with 5 mL of hydrosol and was placed in a shaker for 24 h. According to spectroscopy, almost the whole amount of the NPs (more than 95%) was adsorbed by the support. After that, to attach the particles to the support, to destruct, and to remove the organic component from the surface of catalysts, the samples were heated in air at 573 K for 2 h. The calcination temperature of 573 K is set to avoid carbonization of the catalyst surface during the decomposition of organic compounds. Before carrying out the catalytic experiment, the samples were calcined at 593 K in a high-vacuum furnace with evacuation using a differential pumping system for 2 h to a pressure (3–5) × 10^−6^ Torr.

### 3.4. Catalytic Procedure

The kinetics of the reactions were investigated under static conditions at a reaction mixture pressure of 0.5 Torr. At this condition, the influence of diffusion is excluded, and true kinetic dependences are observed. Analysis of the gas mixture was carried out continuously by thermal conductivity. The experimental technique is described in more detail in previous works [21,52,53].

### 3.5. Characterization

#### 3.5.1. UV-Vis Spectroscopy

Synthesis and impregnation of gold NPs were controlled by UV-Vis spectroscopy. Optical spectra were measured using a Cary 100 Scan spectrophotometer (Varian Inc., Utrecht, The Netherlands) equipped with a Peltier cell. The measurements were carried out in a quartz cell (Hellma, Müllheim, Germany) with an optical path length of 10 mm at 20 °C.

#### 3.5.2. Dynamic Light Scattering

The hydrodynamic size and the ζ-potential of the silver nanoparticles were determined by dynamic light scattering on a Delsa Nano C instrument (Beckman Coulter, Inc., Brea, CA, USA). The wavelength of the scattered laser radiation was λ = 658 nm. Measurements were carried out in a quartz cell with an optical path length of 10 mm. Before starting the measurement, the solution was thermostated at 25 °C.

#### 3.5.3. Electron Microscopy

The nanoparticle size and shape were determined by transmission electron microscopy (TEM) on a JEM-2100 microscope (Jeol, Tokyo, Japan) operating at an accelerating voltage of 200 kV. The samples for TEM were prepared by putting a drop of nanoparticle dispersions on a copper grid (Ted Pella, Redding, CA, USA, 400 mesh) coated ultrathin carbon (<3 nm) on carbon lacey support film followed by drying at ambient temperature. Selected area electron diffraction (SAED) patterns were obtained to determine the crystal structure of the synthesized particles.

Scanning electron microscopy (SEM) images were recorded with a LYRA3 microscope (Tescan, Brno, Czech Republic) in the secondary electron mode using an InBeam detector at an accelerating voltage of 5–10 kV, equipped with an energy-dispersive X-ray (EDX) spectrometer. The elemental composition of the samples was determined by EDX spectroscopy. The samples for SEM were prepared by putting a catalyst onto a highly oriented pyrolytic graphite (HOPG) substrate. The SEM and EDX images are presented in Figures S3 and S4, respectively.

Size distributions were plotted using the measurements of no less than 100 randomly selected nanoparticles. TEM images were measured manually using the free software ImageJ (Version 1.54k) (https://imagej.net accessed on 24 September 2024).

## 4. Conclusions

The coordination environment of the central metal atom largely determines the catalytic activity of metal/metal oxide catalysts. A study of the catalytic activity of gold atoms in the corners and at the edges of gold nanoparticles in the H2-D2 isotope exchange reaction confirms this important position in catalysis and provides a quantitative assessment of the coordination influence. The specific activity at 77 K was found to increase with increasing nanoparticle size from 0.7 to 40 nm deposited on γ-Al_2_O_3_ carrier, while it increases sharply for particle size less than 3 nm. The results indicate the presence of two types of catalysis centers for the hydrogen isotope exchange reaction on the surface of gold nanoparticles, which are due to low-coordination atoms at the edges and corners. The catalytic activity of gold atoms at the corners is about 40 times higher than that of gold atoms at the edges. The developed model, which takes into account the presence of such centers on the surface and their share in the nanoparticle size, adequately describes the experimental data. It can be assumed that the difference between the catalytic sites of gold nanoparticles is not limited to their activity with respect to dissociative adsorption of hydrogen, but can also manifest itself in the selectivity of some other reactions. It is likely that a noticeable change in the electronic state of nanosized gold particles in the range of 1–2 nm may indicate a transition to the nonmetallic phase, and its structural sensitivity is due to the quantum size effect. On the other hand, gold particles larger than 2–3 nm are already metallic in nature and can demonstrate catalytic activity and selectivity in carrying out other reactions of organic synthesis.

## Figures and Tables

**Table 1:** Specific surface of Au@Al_2_O_3_ catalysts and activation energy in high- and low-temperature regions of the hydrogen isotope exchange reaction.

*d*, nm	*S*_H_, m^2^ g^−1^	log*K*_sp,ave_, Molecules s^−1^ cm^−2^	*E*_a_^HT^, kJ mol^−1^	*E*_a_^LT^, kJ mol^−1^
0.7 ± 0.2	0.11 ± 0.01	14.37	9.6 ± 2.1	0.16 ± 0.39
0.9 ± 0.3	0.09 ± 0.01	14.27	14.3 ± 1.1	0.05 ± 0.18
1.0 ± 0.3	0.13 ± 0.02	14.12	10.8 ± 1.7	−0.9 ± 0.6
1.1 ± 0.3	0.13 ± 0.02	14.15	9.6 ± 1.7	−0.76 ± 0.58
4.6 ± 0.8	0.11 ± 0.02	13.1	8.5 ± 1.9	−0.69 ± 0.92
7.4 ± 1.1	0.12 ± 0.03	12.75	12.5 ± 2.8	1.1 ± 0.6
14.4 ± 2.2	0.10 ± 0.01	12.19	23.4 ± 8.8	1.7 ± 0.6
19.4 ± 4.2	0.08 ± 0.02	11.95	34.4 ± 7.8	2.1 ± 0.1
20.5 ± 4.5	0.09 ± 0.02	11.96	35.7 ± 11.3	1.8 ± 0.3
28.3 ± 3.1	0.08 ± 0.02	11.93	42.1 ± 9.3	1.5 ± 0.4
40.1 ± 5.4	0.07 ± 0.01	11.48	36 ± 13.3	2.0 ± 0.7

## Data Availability

The authors confirm that the data supporting the findings of this study are available within the article and its supplementary materials.

## Supplementary Materials

The following supporting information can be downloaded at: https://www.mdpi.com/article/10.3390/ijms252212022/s1.

## References

[1] Sun X., Li F., Shi J., Zheng Y., Su H., Sun L., Peng S., Qi C. (2019). Gold nanoparticles supported on MgOx-Al2O3 composite oxide: An efficient catalyst for selective hydrogenation of acetylene. Appl. Surf. Sci..

[2] Holz J., Pfeffer C., Zuo H., Beierlein D., Richter G., Klemm E., Peters R. (2019). In Situ Generated Gold Nanoparticles on Active Carbon as Reusable Highly Efficient Catalysts for a C_sp3_−C_sp3_ Stille Coupling. Angew. Chem. Int. Ed..

[3] Zhao J., Ge L., Yuan H., Liu Y., Gui Y., Zhang B., Zhou L., Fang S. (2019). Heterogeneous gold catalysts for selective hydrogenation: From nanoparticles to atomically precise nanoclusters. Nanoscale.

[4] Chen J., Zhang J., Zhu D., Li T. (2019). One-pot synthesized porphyrin-based polymer supported gold nanoparticles as efficient catalysts for alkyne hydration and alcohol oxidation in water. Gold Bull..

[5] Tran T.D., Nguyen M.T.T., Le H.V., Nguyen D.N., Truong Q.D., Tran P.D. (2018). Gold nanoparticles as an outstanding catalyst for the hydrogen evolution reaction. Chem. Commun..

[6] Lin C., Tao K., Hua D., Ma Z., Zhou S. (2013). Size Effect of Gold Nanoparticles in Catalytic Reduction of p-Nitrophenol with NaBH4. Molecules.

[7] Demirel-Gülen S., Lucas M., Claus P. (2005). Liquid phase oxidation of glycerol over carbon supported gold catalysts. Catal. Today.

[8] Qian K., Luo L., Bao H., Hua Q., Jiang Z., Huang W. (2013). Catalytically active structures of SiO_2_-supported Au nanoparticles in low-temperature CO oxidation. Catal. Sci. Technol..

[9] Meyer R., Lemire C., Shaikhutdinov S.K., Freund H.J. (2004). Surface chemistry of catalysis by gold. Gold Bull..

[10] Gatin A., Grishin M., Dokhlikova N., Ozerin S., Sarvadii S., Kharitonov V., Shub B. (2019). Effect of Size on Hydrogen Adsorption on the Surface of Deposited Gold Nanoparticles. Nanomaterials.

[11] Lin S.D., Bollinger M., Vannice M.A. (1993). Low temperature CO oxidation over Au/TiO_2_ and Au/SiO_2_ catalysts. Catal. Lett..

[12] Fujitani T., Nakamura I., Akita T., Okumura M., Haruta M. (2009). Hydrogen Dissociation by Gold Clusters. Angew. Chem. Int. Ed..

[13] Valden M., Lai X., Goodman D.W. (1998). Onset of Catalytic Activity of Gold Clusters on Titania with the Appearance of Nonmetallic Properties. Science.

[14] Chen, Cai Y., Yan Z., Goodman D.W. (2006). On the Origin of the Unique Properties of Supported Au Nanoparticles. J. Am. Chem. Soc..

[15] Okazaki K., Ichikawa S., Maeda Y., Haruta M., Kohyama M. (2005). Electronic structures of Au supported on TiO_2_. Appl. Catal. A Gen..

[16] Liang C., Cheong J.Y., Sitaru G., Rosenfeldt S., Schenk A.S., Gekle S., Kim I., Greiner A. (2022). Size-Dependent Catalytic Behavior of Gold Nanoparticles. Adv. Mater. Interfaces.

[17] De S.K., Mondal S., Sen P., Pal U., Pathak B., Rawat K.S., Bardhan M., Bhattacharya M., Satpati B., De A. (2018). Crystal-defect-induced facet-dependent electrocatalytic activity of 3D gold nanoflowers for the selective nanomolar detection of ascorbic acid. Nanoscale.

[18] Bus E., Miller J.T., van Bokhoven J.A. (2005). Hydrogen Chemisorption on Al 2 O 3 -Supported Gold Catalysts. J. Phys. Chem. B.

[19] Abkhalimov E.V., Boeva O.A., Odintzov A.A., Solovov R.D., Zhavoronkova K.N., Ershov B.G. (2020). The H2-D2 exchange reaction catalyzed by gold nanoparticles supported on γ-Al2O3: Effect of particle size on the reaction rate. Catal. Commun..

[20] Boeva O.A., Ershov B.G., Zhavoronkova K.N., Odintsov A.A., Solovov R.D., Abkhalimov E.V., Evdokimenko N.D. (2015). Catalytic properties of gold nanoparticles in H2—D2 exchange and ortho—Para hydrogen conversion. Dokl. Phys. Chem..

[21] Boeva O.A., Odintzov A.A., Solovov R.D., Abkhalimov E.V., Zhavoronkova K.N., Ershov B.G. (2017). Low-temperature ortho–para hydrogen conversion catalyzed by gold nanoparticles: Particle size does not affect the rate. Int. J. Hydrogen Energy.

[22] Schimpf S., Lucas M., Mohr C., Rodemerck U., Brückner A., Radnik J., Hofmeister H., Claus P. (2002). Supported gold nanoparticles: In-depth catalyst characterization and application in hydrogenation and oxidation reactions. Catal. Today.

[23] Van Hardeveld R., Hartog F. (1969). The statistics of surface atoms and surface sites on metal crystals. Surf. Sci..

[24] Boccuzzi F., Cerrato G., Pinna F., Strukul G. (1998). FTIR, UV−Vis, and HRTEM Study of Au/ZrO_2_ Catalyst: Reduced Reactivity in the CO−O_2_ Reaction of Electron-Deficient Gold Sites Present on the Used Samples. J. Phys. Chem. B.

[25] Ershov B.G., Abkhalimov E.V., Solovov R.D., Roldughin V.I. (2016). Gold nanoparticles in aqueous solutions: Influence of size and pH on hydrogen dissociative adsorption and Au(III) ion reduction. Phys. Chem. Chem. Phys..

[26] Zaera F. (2017). The Surface Chemistry of Metal-Based Hydrogenation Catalysis. ACS Catal..

[27] Gentry N.E., Kurimoto A., Cui K., Cleron J.L., Xiang C.M., Hammes-Schiffer S., Mayer J.M. (2024). Hydrogen on Colloidal Gold Nanoparticles. J. Am. Chem. Soc..

[28] Ishida R., Hayashi S., Yamazoe S., Kato K., Tsukuda T. (2017). Hydrogen-Mediated Electron Doping of Gold Clusters As Revealed by in Situ X-ray and UV-vis Absorption Spectroscopy. J. Phys. Chem. Lett..

[29] Ershov B.G., Roldughin V.I., Abkhalimov E.V., Solovov R.D., Dement’eva O.V., Rudoy V.M. (2014). The effects of hydrogen and ph on plasmon absorption of gold hydrosol. Electrochemical reactions on nanoelectrodes. Colloid J..

[30] Haruta M. (2003). When Gold Is Not Noble: Catalysis by Nanoparticles. Chem. Rec..

[31] Chen L., Wang Q., Xiong L. (2017). Molecular dynamics study on structure stability, lattice variation, and melting behavior of silver nanoparticles. J. Nanoparticle Res..

[32] Geng G., Chen P., Guan B., Liu Y., Yang C., Wang N., Liu M. (2017). Sheetlike gold nanostructures/graphene oxide composites via a one-pot green fabrication protocol and their interesting two-stage catalytic behaviors. RSC Adv..

[33] Lin T.H., Lin C.W., Liu H.H., Sheu J.T., Hung W.H. (2011). Potential-controlled electrodeposition of gold dendrites in the presence of cysteine. Chem. Commun..

[34] Schrinner M., Polzer F., Mei Y., Lu Y., Haupt B., Ballauff M., Göldel A., Drechsler M., Preussner J., Glatzel U. (2007). Mechanism of the Formation of Amorphous Gold Nanoparticles within Spherical Polyelectrolyte Brushes. Macromol. Chem. Phys..

[35] Bond G.C. (2016). Hydrogenation by gold catalysts: An unexpected discovery and a current assessment. Gold Bull..

[36] Doronin S.V., Dokhlikova N.V., Grishin M.V. (2022). Descriptor of catalytic activity nanoparticles surface: Atomic and molecular hydrogen on gold. Mol. Catal..

[37] Janssens T.V.W., Clausen B.S., Hvolbæk B., Falsig H., Christensen C.H., Bligaard T., Nørskov J.K. (2007). Insights into the reactivity of supported Au nanoparticles: Combining theory and experiments. Top. Catal..

[38] Haruta M., Tsubota S., Kobayashi T., Kageyama H., Genet M.J., Delmon B. (1993). Low-Temperature Oxidation of CO over Gold Supported on TiO_2_, α-Fe_2_O_3_, and Co_3_O_4_. J. Catal..

[39] Lyalin A., Taketsugu T. (2011). A computational investigation of H2 adsorption and dissociation on Au nanoparticles supported on TiO_2_ surface. Faraday Discuss..

[40] Svalova A.I., Stishenko P.V. (2016). Distribution of Active Site Types on Au Nanoparticles with Different Structures: Study of Thermal Dependence. Procedia Eng..

[41] Morozov P.A., Ershov B.G., Abkhalimov E.V., Dement’eva O.V., Filippenko M.A., Rudoy V.M., Roldughin V.I. (2012). The effect of ozone on plasmon absorption of gold hydrosols. Quasi-metal and metal nanoparticles. Colloid J..

[42] Logunov S.L., Ahmadi T.S., El-Sayed M.A., Khoury J.T., Whetten R.L. (1997). Electron Dynamics of Passivated Gold Nanocrystals Probed by Subpicosecond Transient Absorption Spectroscopy. J. Phys. Chem. B.

[43] Guan W., Cheng W., Pei S., Chen X., Yuan Z., Lu C. (2024). Probing Coordination Number of Single-Atom Catalysts by d-Band Center-Regulated Luminescence. Angew. Chem. Int. Ed..

[44] Mohr C., Claus P. (2001). Hydrogenation Properties of Supported Nanosized Gold Particles. Sci. Prog..

[45] Che M., Bennett C.O. (1989). The Influence of Particle Size on the Catalytic Properties of Supported Metals. Advances in Catalysis.

[46] Sarvadii S.Y., Gatin A.K., Dokhlikova N.V., Kharitonov V.A., Ozerin S.A., Doronin S.V., Grishin M.V., Shub B.R. (2021). Hydrogenation of HOPG-Supported Gold Nanoparticles: Surface or Volume?. Crystals.

[47] Odintsov A.A., Revina A.A., Zhavoronkova K.N., Boeva O.A. (2016). Catalytic Properties of Gold Nanoparticles Prepared in Reverse Micelles. Prot. Met. Phys. Chem. Surfaces.

[48] Egorova E., Revina A. (2000). Synthesis of metallic nanoparticles in reverse micelles in the presence of quercetin. Colloids Surfaces A Physicochem. Eng. Asp..

[49] Solovov R.D., Ershov B.G. (2014). Preparation of palladium nanoparticles with desired sizes in aqueous solutions. Colloid J..

[50] Turkevich J., Stevenson P.C., Hillier J. (1951). A study of the nucleation and growth processes in the synthesis of colloidal gold. Discuss. Faraday Soc..

[51] Aswathy Aromal S., Philip D. (2012). Facile one-pot synthesis of gold nanoparticles using tannic acid and its application in catalysis. Phys. E Low-Dimens. Syst. Nanostructures.

[52] Boeva O., Antonov A., Zhavoronkova K. (2021). Influence of the nature of IB group metals on catalytic activity in reactions of homomolecular hydrogen exchange on Cu, Ag, Au nanoparticles. Catal. Commun..

[53] Boeva O., Kudinova E., Vorakso I., Zhavoronkova K., Antonov A. (2022). Bimetallic gold-copper nanoparticles in the catalytic reaction of deuterium-hydrogen exchange: A synergistic effect. Int. J. Hydrogen Energy.

