# Supplementary material for: Difference in the Catalytic Activity of Atoms in the Corners and at the Edges of Gold Nanoparticles: Hydrogen Isotope Exchange Reaction
Source: Int J Mol Sci. 2024 Nov 8;25(22):12022. doi: 10.3390/ijms252212022 (PMC11594137; doi:10.3390/ijms252212022)
Supplement: Supplementary file 1 [file ijms-25-12022-s001.zip › ijms-3256526-supplementary.pdf]

Supplimentery materials

Difference in the catalytic activity of atoms in the corners and at the edges of gold nanoparticles: hydrogen isotope exchange reaction

Evgeny Abkhalimov,* Boris Ershov

A.N. Frumkin Institute of Physical chemistry and Electrochemistry, Russian Academy of Science, Leninsky pr. 31-4, Moscow, 119071, Russia. *E-mail: abkhalimov@ipc.rssi.ru

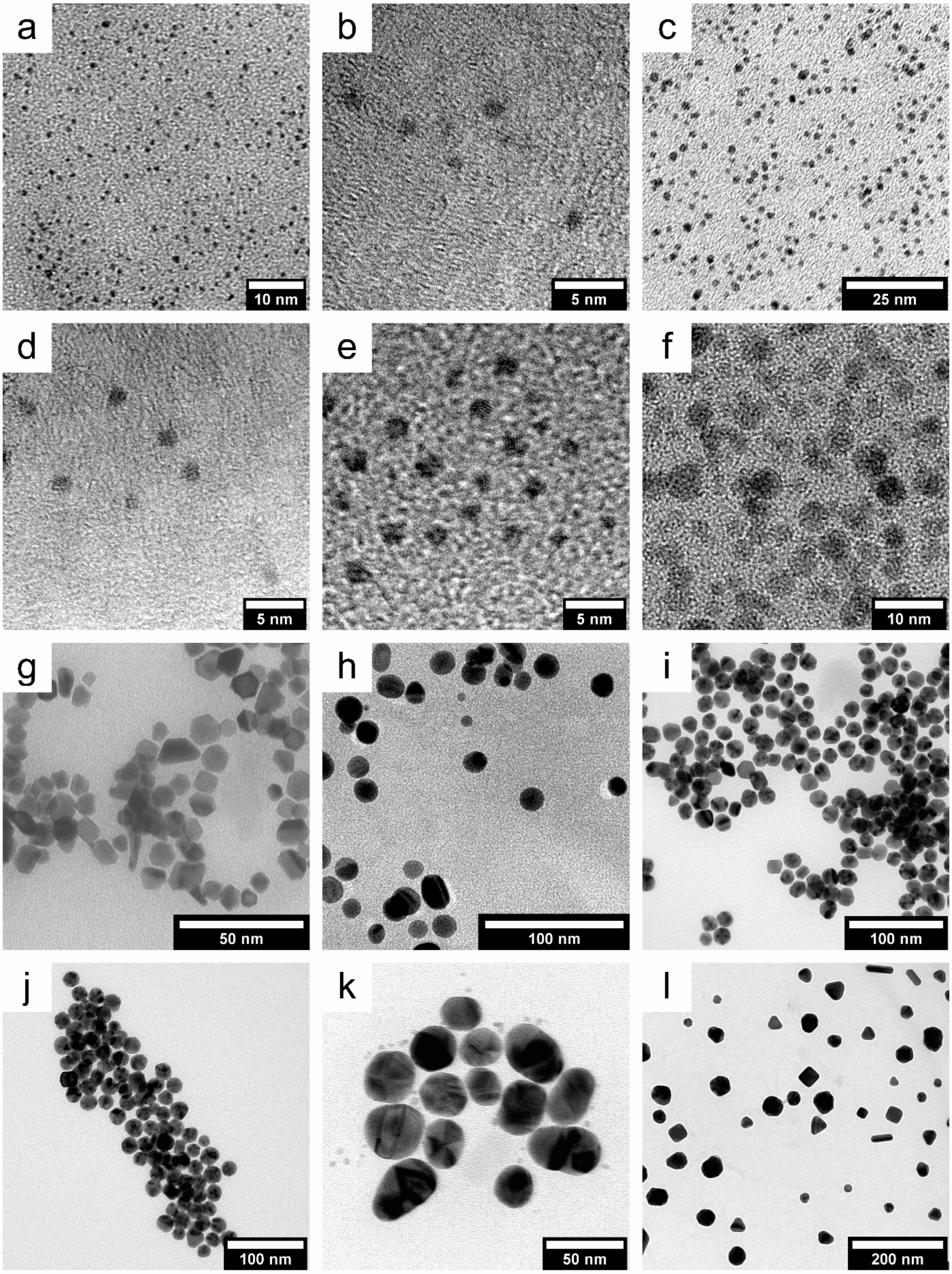

Figure S1. TEM images with different sizes, nm: a – 0.7; b – 0.9; c –1.0; d – 1.1; e – 1.4; f – 4.6; g – 7.4; h – 14.4; i – 19.4; j – 20.5; k – 28.3; l – 40.1.

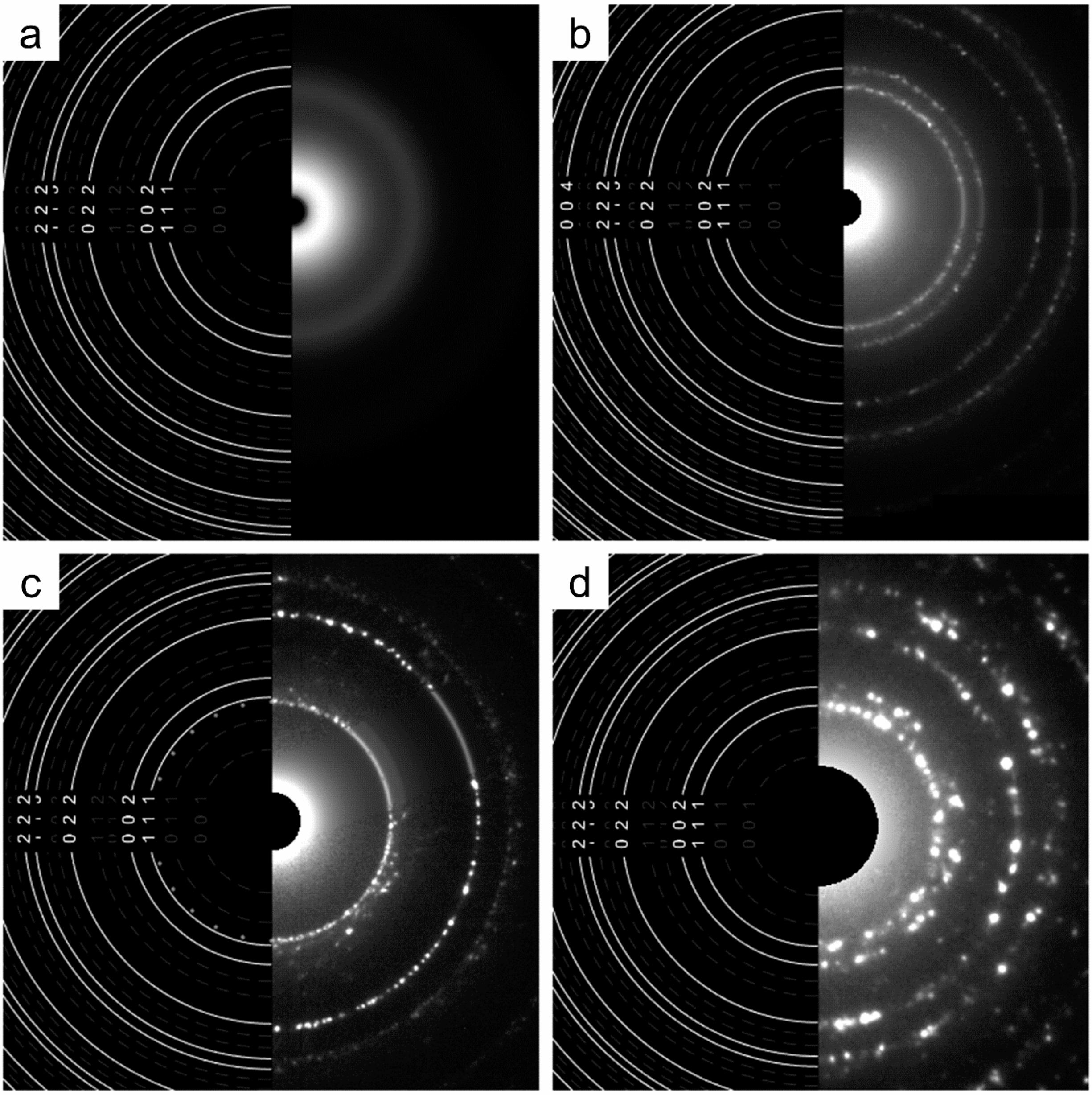

Figure S2. SAED pattern images: a – 1.4 nm; b – 7.4 nm; c – 20.5 nm; d – 40.1 nm.

Table S1. Lattice parameters of Au NPs[a].

d, nm	Lattice spacing, Å				Lattice parameter, Å
	111	200	220	311	
1.4	2.38	-	-	-	4.12
7.4	2.35	2.03	1.43	1.22	4.06
14.4	2.37	2.06	1.47	1.23	4.07
40.1	2.33	2.03	1.43	1.21	4.04
Theor., Å	2.36	2.04	1.44	1.23	4.08

[a] calculated from Fig. S2.

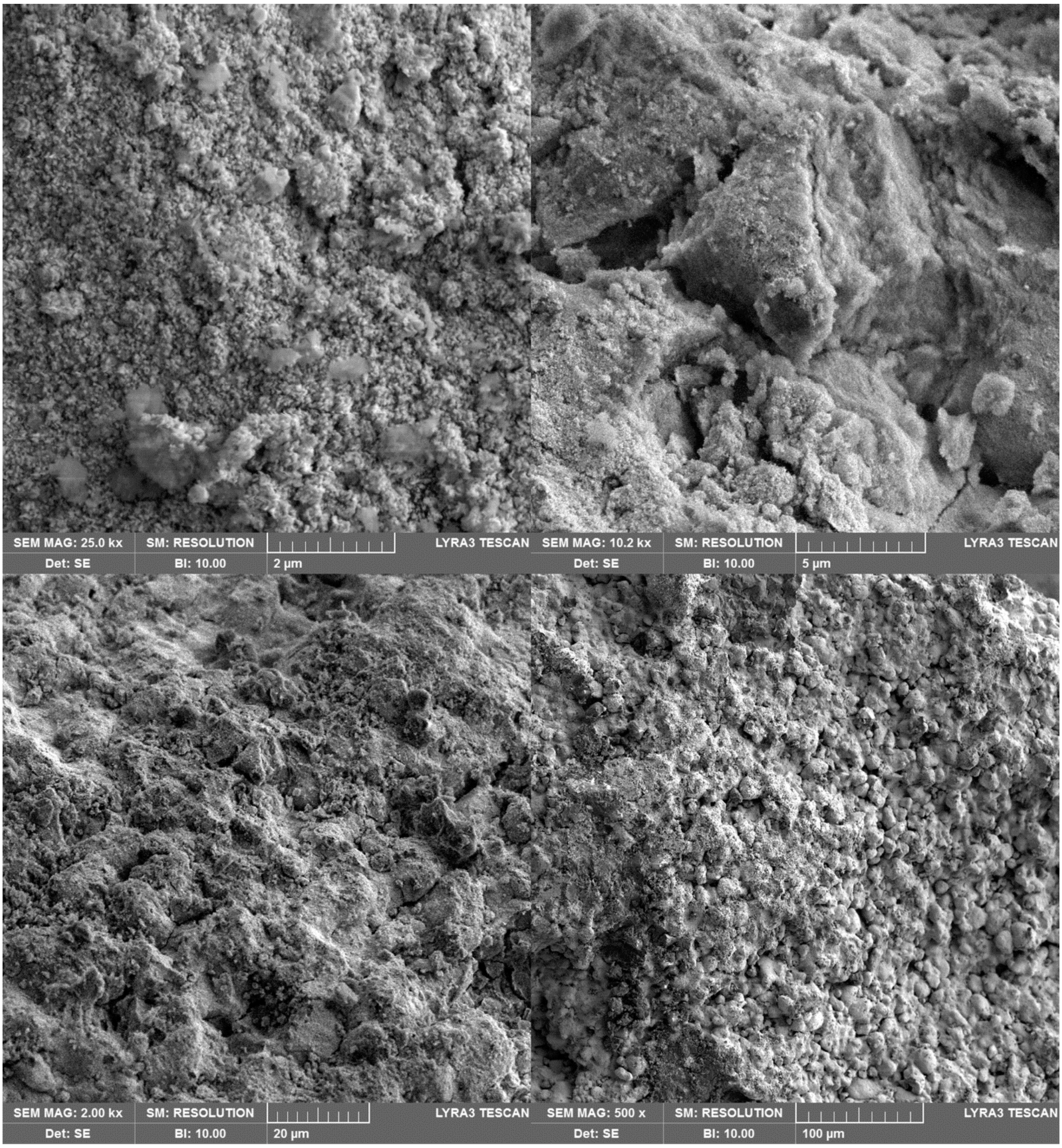

Figure S3. SEM images of Au@Al_2O_3 catalyst at different magnifications.

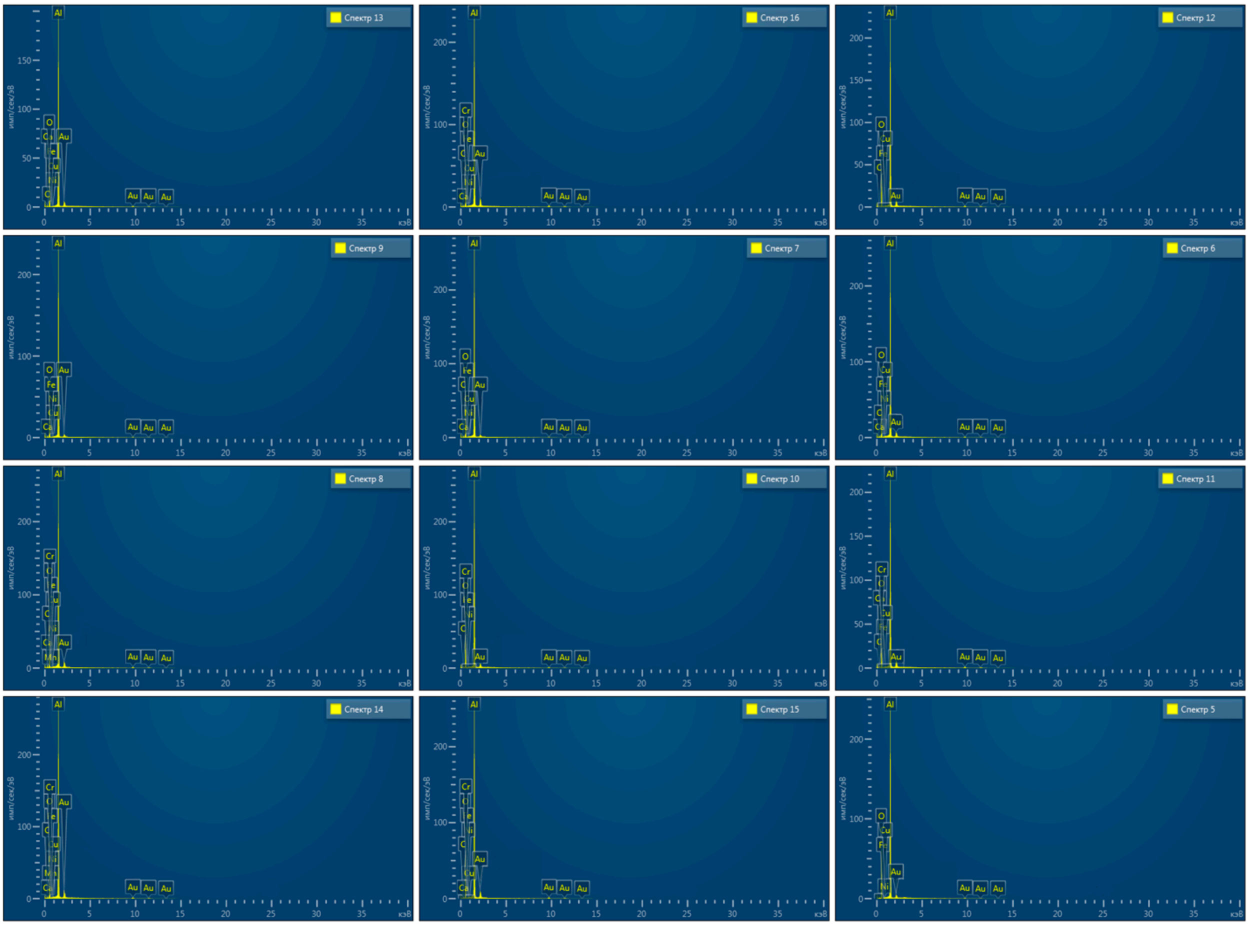

Figure S4. EDX spectra of $Au@Al_2O_3$ catalyst: a – 0.7 nm; b – 0.9 nm; c –1.0 nm; d – 1.1 nm; e – 1.4 nm; f – 4.6 nm; g – 7.4 nm; h – 14.4 nm; i – 19.4 nm; j – 20.5 nm; k – 28.3 nm; l – 40.1 nm.
